# Referrals for vulval appearance concerns to a specialist children and young person’s female genital cutting/mutilation (FGC/M) clinic: a ten-year retrospective analysis

**DOI:** 10.1038/s41443-025-01188-2

**Published:** 2025-10-16

**Authors:** Hazel Isabella Learner, Sakaria Ali, Nikita Deegan, Deborah Hodes

**Affiliations:** 1 Department of Women’s Health, University College London Hospitals, 2nd Floor North Wing, 250 Euston Road, London, NW1 2PG UK; 2 Department of Paediatrics, University College London Hospitals, 5th Floor North Wing, 250 Euston Road, London, NW1 2PG UK; 3 Department of Obstetrics and Gynaecology, The Rotunda Hospital, Parnell Square Dublin 1, DO1 P5W9, Dublin, Ireland; 4 Department of Paediatrics (Ret.), University College London Hospitals, 5th Floor North Wing, 250 Euston Road, London, NW1 2PG UK

**Keywords:** Diagnosis, Anatomy, Risk factors

## Abstract

The diagnosis of female genital cutting/mutilation (FGC/M) in children is complex, as findings may be subtle or mimic normal vulval variation. England and Wales safeguarding guidance advises referral of any signs of FGC/M, regardless of the observer’s background. We hypothesised that most children referred to a specialist clinic for concerns about vulval appearance would not have undergone FGC/M, reflecting gaps in anatomical knowledge and limitations of current guidance. A retrospective review of electronic medical records for all children (0–17years) seen from October 2014 to March 2025 was undertaken. The primary outcome was the proportion of appearance-related referrals. Secondary endpoints included confirmed finding of FGC/M, family history of FGC/M, vulval findings, and whose concerns triggered the referral. Among 291 referrals, 129/291 (44.3%) were confirmed to have undergone FGC/M. Forty-two cases (42/291 (14.4%)) were triggered for appearance concerns (median age 4 y IQR 2–11). FGC/M was verified in just one of these 42 children (1/42 (2.4%)). There was a family history of FGC/M in 14/42 (33.3%). Thirty-four (34/42 (81.0%)) had typical anatomy and six (6/42 (14.3%)) had common paediatric vulval conditions. Non-clinicians generated 26/42 referrals (61.9%). Updated guidance is needed to balance vigilance in safeguarding with a more nuanced, informed and child-centered approach.

## Introduction

Female genital cutting/mutilation (FGC/M) is a harmful practice and it is recognised as both a public health and a human rights issue. The practice is performed in many communities globally with estimates that over 230 million women and girls have genital cutting for different sociocultural reasons [1].

The term female genital mutilation (FGM) is controversial for its western bias, reductiveness, and the stigmatization of communities and individuals affected [2]. It is increasingly common to use FGC/M to focus on individual trauma whilst acknowledging the importance of advocating against the practice.

There are estimated to be over one hundred thousand women who have undergone FGC/M living in the United Kingdom (UK) [3] and 600,000 in Europe [4]. National data collection such as the FGM information sharing system and the NHS Enhanced Dataset have been recently developed [5], but it is not yet clear how many UK resident children have undergone FGC/M. There is no firm evidence to confirm the extrapolations from migration data that nearly ten thousand children in the UK aged 0–14years, from FGC/M affected communities, have undergone FGC/M or are at risk of FGC/M [3, 6, 7]. A significant number of legal and educational measures have been introduced in the UK with the aim of protecting children from FGC/M [8–10]. The mandatory reporting duty included in the Serious Crime Act of 2015 requires regulated professionals in health, education and social care in England and Wales to report to the police all children under 18 years old who disclose FGC/M or have physical evidence of FGC/M [11].

There is a paucity of studies that have examined referrals for suspected FGC/M in children. Johnsdotter identified 43/61 children were found not to have undergone FGC/M on genital examinations as part of police investigations in Sweden, with the majority of these 43 children found to have normal genitalia [12]. This study aimed to consider the hypothesis that the majority of children and young people referred to a UK specialist clinic with concerns about vulval appearances would not have undergone FGC/M. Instead, we anticipated that these referrals would largely reflect normal anatomical variation or benign paediatric vulval conditions. This hypothesis is grounded in the recognition that healthcare and non-healthcare professionals may have limited exposure to the spectrum of normal vulval variation – particularly in children [13, 14] and that current safeguarding guidance, while appropriately prioritising vigilance, may inadvertently encourage over-referrals.

## Materials and methods

This was a retrospective study of children seen at a specialist FGC/M children and young people’s clinic. The study’s primary outcome measure was the proportion of vulval appearance related referrals, with secondary outcomes of whether these children referred were confirmed to have undergone FGC/M, had a family history of FGC/M, the event and whose concerns triggered the referral, and vulval findings in clinic. The study reviews a dedicated multi-disciplinary service led by a consultant paediatrician and a consultant paediatric and adolescent gynaecologist. The clinical team include psychology and a clinical specialist nurse. The service was the first dedicated children and young persons’ FGC/M clinic and is recognised as the only national referral centre in the UK. [7]

The clinic was established in September 2014 and the study period reviews ten years of clinic data up to March 2025. All new clinic appointments for children aged 17 and under were identified through the clinic’s governance database and included in the study. Demographic information, reason for referral, family history, medical history and examination findings for all these children were extracted from electronic medical records, including clinic proformas and consultation reports.

Our clinic supports bodily autonomy and therefore only examined children with informed consent from themselves or their parent *(*or rarely the social worker where appropriate*)*. The genital examination was performed in the frog-legged position with a colposcope for light, magnification, and photo-documentation. The examination is performed jointly by two senior clinicians with expertise in paediatric and adolescent genital examination of children and the findings of FGC/M. Evidence of FGC/M was taken as genital examination findings consistent with FGC/M or where examination declined when a history from the child and/or parent described FGC/M. Data was recorded in a secure Excel^TM^ spreadsheet (Microsoft 161 Corporation, Seattle, USA) which was used to provide basic statistical measures to describe the above. The local Research Ethics Committee approved the study as a service review, providing all patient identifiable information was removed prior to analysis and publication.

## Results

Over the ten-year period, the clinic saw a total of 303 children and young people. Twelve individuals aged 18 to 20 years were excluded from this study, resulting in a final sample of 291. Of the 291 children and young people included in the study 44.3% (129/291) had undergone FGC/M. Table 1 shows the reasons for referral.

**Table 1:** Reason for FGC/M clinic referral.

Reason for referral, No. (%)	n = 291
History of previously undergoing FGC/M	142 (48.8)
Family history of FGC/M	42 (14.4)
Vulval appearance concerns	42 (14.4)
Travel concerns	33 (11.3)
Collateral referral because of FGC/M concerns raised in sister	18 (6.2)
Alleged parental plan for, or support of, FGC/M	7 (2.4)
Behavior concerns	5 (1.7)
Asylum application - No history of FGC/M in child	2 (0.7)

*FGC/M* (female genital cutting / mutilation)

The majority of children and young people confirmed to have undergone FGC/M were referred with a history of FGC/M disclosed by themselves or their family (123/129 (95.3%)).

There were 42/291 (14.4%) children and young people referred with vulval appearance concerns; with numbers of referrals per year ranging from 0–7, with no clear temporal trends in referral rates.

Additionally, the siblings in nine of the children and young people where vulval concerns were raised, were also referred to the clinic for an examination. The age, family history and ethnic background of the children and young people referred is shown in Table 2. Family history was unavailable for some of the children in care or who were unaccompanied asylum seekers. Only one referral (1/42 (2.4%)) presented with acute genital injuries- which was the result of FGC/M. There were 5/42 (12%) children placed in foster care by the local authorities. Three of these children were in care for safeguarding issues unrelated to potential FGC/M concerns. Two were in care because of FGC/M concerns, one of these was the child who presented with the acute injuries due to FGC/M. This child presented with bleeding and a haematoma and was found to have absent labia minora. The account of the injury mechanism was not consistent with the clinical findings. The other was a child with learning needs and vulval concerns thought to be FGC/M and was taken into care prior to the referral to the specialist clinic; she was found to have typical vulva on examination. There were 26/42 (61.9%) children referred following vulval concerns raised by individuals not clinically trained (Table 3). Of these 26 children 20/26 (76.9%) were referred to the specialist clinic without a clinician first examining the child. Table four summarises the vulval appearance FGC/M concerns that prompted referral. Approximately two thirds of referrals (25/42 60%) were with non-specific descriptions such as ’unusual appearance’, ‘abnormalities seen’, ‘looks different’, ‘not normal’, ‘suggestive of FGM’. Eight children and young people were referred with previous genital findings of absent labia – with all recognised at specialist examination to have labia present with appearances typical for their age/pubertal status. One young person was referred by the police with a history of labiaplasty and specialist clinical review confirmed previous labiaplasty with reduction of labia minora.

**Table 2:** Patient characteristics.

		All children and young people referred (n = 291)	Referred with vulval appearance concerns (42/291)
**Age**		Median 10years (IQR 6-14)	Median 4years (IQR 2-11)
**FGC/M confirmed**		129/291 (44.3%)	1/42 (2.4%)
**Ethnic background**	African	270/291 (92.8%)	34/42 (81.0%)
	Asian	15/291 (5.2%)	6/42 (14.2%)
	Arabic	1/291 (0.3%)	1/42 (2.4%)
	White-other	5/291 (1.7%)	1/42 (2.4%)
**Family history of FGC/M**	Yes	220/291 (75.6%)	14/42 (33.3%)
	No	48/291 (16.5%)	25/42 (59.6%)
	Unknown	23/291 (7.9%)	3/42 (7.1%)

*FGC/M* (female genital cutting / mutilation)

**Table 3:** Event and individual that prompted referral.

Event raising vulval concerns, No. (%)	n = 42
Nursery helping with changing	16 (38.1)
Genital examination for vulval discomfort	7 (16.7)
School helping with changing	6 (14.3)
Genital examination for urinary symptoms	4 (9.5)
Genital examination for catheterisation	3 (7.1)
Family member helping with changing	2 (4.8)
Foster carer helping with changing	2 (4.8)
Genital examination for acute genital injuries	1 (2.4)
Forensic examination for suspected sexual assault	1 (2.4)
**Clinical examination prior to referral, No. (%)**	**n** = **42**
No clinical examination ahead of referral	20 (47.6)
Primary care: general practice	10 (23.8)
Emergency department	2 (4.8)
Sexual health	2 (4.8)
Paediatrician	2 (4.8)
Sexaual Assault Referral Centre	2 (4.8)
Urology	2 (4.8)
Dermatology	1 (2.4)
Gynaecology	1 (2.4)

*FGC/M* (female genital cutting / mutilation)

All the children referred with vulval appearance concerns and their parents, consented to a genital examination. Specialist clinic review findings are summarized in Table 4. The majority had typical vulval appearances on examination (34/42 (81%)) and six found to have vulval findings typical of common benign vulval conditions. These are common vulval conditions of childhood and are not as a result of, nor linked to FGC/M. None of these six children had a family history of FGC/M. There is a UK government produced a risk assessment tool specifically for health professionals working in England and Wales [9]. This study found no evidence that the risk assessment tool was used to guide referrers as evidenced by their referral letters and copies of strategy minutes where available.

**Table 4:** Vulval concerns and specialist clinic findings.

Referring vulval FGCM concern, No,(%)	n = 42	Previous medical examination (22/42)
Non-specific vulval appearance concerns	25 (59.5)	11 (50.0)
Absent labia	8 (19.0)	4 (18.2)
Closed introitus	5 (11.9)	3 (13.6)
FGC/M as a differential for Lichen Scerlosis	1 (2.4)	1 (4.5)
Acute labial injuries	1 (2.4)	1 (4.5)
Clitoral hood partially absent	1 (2.4)	1 (4.5)
Labiaplasty	1 (2.4)	1 (4.5)
**Specialist FGC/M clinic findings, No. (%)**	**n** = **42**	
Normal examination: typical vulval appearances (in respect to age and pubertal development)	34 (81.0)	15 (68.2)
Labial adhesions	2 (4.8)	2 (9.1)
Vitiligo	2 (4.8)	1 (4.5)
Lichen sclerosus	1 (2.4)	1 (4.5)
Urethral prolapse	1 (2.4)	1 (4.5)
Vulvovaginitis	1 (2.4)	1 (4.5)
Acute injuries consistent with FGC/M	1 (2.4)	1 (4.5)

*FGC/M* (female genital cutting / mutilation)

## Discussion

This study presents over 10 years of data from a specialist children and young people’s FGC/M service. This is a national service, based in London, as such this cohort is likely representative of referrals for vulval appearance concerns nationally. The number of health professionals with expertise in identifying FGC/M in children and young people is limited. This study supports the need for improved training for paediatricians and frontline clinicians in both normal vulval development and FGC/M findings. Delays in arranging medical assessments for FGC/M can cause unnecessary distress for children and their families. One child in this study was placed in foster care based on the non-clinical observations highlighting the significant consequences of misinterpretations. The physical signs of FGC/M are highly variable, and WHO FGM Type 4 (Table 5) can be difficult to detect even with expert examination [15]. The prepubertal vulva typically has small labia minora, which with oestrogen levels rising during puberty the labia minora thicken and lengthen [16]. The development of the vulva with puberty is poorly understood as demonstrated by the absence of vulval development described in Tanner’s staging of puberty [17]. This lack of knowledge can result in the mistaken assumption that labia are absent or abnormal [18]. This study demonstrates a lack of awareness of the variation of vulval development. Resources such as the recently published Atlas on Female Genital Mutilation/Cutting among children and adolescents offers valuable guidance, illustrating vulval variation, vulval changes throughout childhood and variation in FGC/M findings [18]. Twenty referrals in this study were triggered by vulva assessments from non-clinicians. We recommend that in the absence of other safeguarding concerns, a prompt medical assessment should be prioritised. This approach allows timely, informed decision-making and is less likely to cause unnecessary escalation and additional psychological distress. Medical conditions affecting the vulva were underdiagnosed in this cohort. There were six children and young people without a family history of FGC/M were referred with signs of vulval dermatosis to a safeguarding FGC/M clinic rather than for a gynecology or dermatology medical assessment. This finding underscores how assumptions about ethnicity or culture can influence reasoning and referral pathways. This represents another dimension of the stigmatization and poor care that can be experienced by communities affected by FGC/M [19] can be extended to people of colour who do not have a family or cultural history of FGC/M.

**Table 5:** WHO FGM Classification [1].

WHO FGM classification
**Type I**. This is the partial or total removal of the clitoral glans (the external and visible part of the clitoris, which is a sensitive part of the female genitals), and/or the prepuce/clitoral hood (the fold of skin surrounding the clitoral glans).
**Type II**. This is the partial or total removal of the clitoral glans and the labia minora (the inner folds of the vulva), with or without removal of the labia majora (the outer folds of skin of the vulva).
**Type III**. Also known as infibulation, this is the narrowing of the vaginal opening through the creation of a covering seal. The seal is formed by cutting and repositioning the labia minora, or labia majora, sometimes through stitching, with or without removal of the clitoral prepuce/clitoral hood and glans.
**Type IV**. This includes all other harmful procedures to the female genitalia for non-medical purposes, e.g., pricking, piercing, incising, scraping and cauterizing the genital area.

*FGCM* (female genital mutilation)

Conversely, the only confirmed case of FGC/M in this cohort found to have findings consistent with recent FGC/M had no known family history of FGC/M. This serves as a reminder that safeguarding decisions must remain child-centered and evidence-informed, not solely based on cultural and ethnic background. FGC/M should be considered particularly where there are acute injuries which do not appear consistent with mechanism of injury.

The generalisability of our findings may be limited by the UK’s specific legal and healthcare context. Research suggests that most children seen with FGC/M in the UK were subjected to this prior to UK entry [6]. There is increasing international evidence that shows in a new social context, pressures on immigrant communities to perform FGC/M are reduced [19–21]. There is growing optimism that this trend applies to the UK [6, 22].

Comparatively, in countries with higher FGC/M prevalence, where the practice is culturally embedded, responses to eradicating FGC/M typically focus on public health campaigns with community engagement, economic incentives for traditional practitioners to find an alternative income, and legal reform [23]. In contrast low-prevalence settings prioritise safeguarding and legal compliance. However, without adequate training safeguarding measures can inadvertently cause harm. Our findings suggest that current approaches in the UK may risk reinforcing racialised assumptions and stigma of FGC/M perpetuating cultural biases and the othering of people of colour [24]. Additionally, our study highlights the overlap of labiaplasty with WHO FGM classification and female genital cosmetic surgery (FGCS). Labiaplasty - commonly performed in adults and typically involving excision of labia minora tissue - closely mirrors WHO FGM Type 2. The England and Wales legal definition of FGM is not limited by the age of the individual affected (ie. no distinction between children or adult women in the law). Professional bodies have advised against FGCS in minors [25, 26] and the Royal College of Obstetricians and Gynaecologists (RCOG) affirmed this stance in a 2013 position statement [27]. This statement however is no longer available on the website and no updated guidance has been issued to clarify which aspect of the previous statement the RCOG no longer agrees with.

### Study limitations

This study has several limitations. As a retrospective service evaluation, it relies on the completeness and accuracy of clinical documentation, although the use of electronic medical records and a structured clinic proforma supports consistency. Given the structure of healthcare throughout the UK through regional differences in awareness of FGC/M and access to expertise which may limit generalisability of these results. Lastly, the study does not capture the broader psychosocial impact on families subjected to safeguarding investigations and police involvement based on mistaken impression of a child’s vulval appearances. Prospective research including caregiver and child experiences would provide further valuable insight.

## Conclusion

We conclude that urgent attention therefore should be given to update professional guidance to ensure to help identify those children at risk of FGC\M without stigmatising communities who do not support FGC/M.

This study supports the need for urgent change in the multiagency referral pathways to include early paediatric review once a case of suspected FGC/M has been identified.

## Data Availability

The datasets generated during and/or analysed during the current study are not publicly available due to the small sample group and potential to identify. Limited datasets are available from the corresponding author on reasonable request.
